# Layered Double Hydroxide-Modified Organic Electrochemical Transistor for Glucose and Lactate Biosensing

**DOI:** 10.3390/s20123453

**Published:** 2020-06-18

**Authors:** Isacco Gualandi, Marta Tessarolo, Federica Mariani, Danilo Arcangeli, Luca Possanzini, Domenica Tonelli, Beatrice Fraboni, Erika Scavetta

**Affiliations:** 1 Dipartimento di Chimica Industriale ‘Toso Montanari’, Università di Bologna, Viale Risorgimento 4, 40136 Bologna, Italy; isacco.gualandi2@unibo.it (I.G.); federica.mariani8@unibo.it (F.M.); danilo.arcangeli@studio.unibo.it (D.A.); domenica.tonelli@unibo.it (D.T.); 2 Dipartimento di Fisica e Astronomia, Università di Bologna, Viale Berti Pichat 6/2, 40127 Bologna, Italy; marta.tessarolo3@unibo.it (M.T.); luca.possanzini2@unibo.it (L.P.); beatrice.fraboni@unibo.it (B.F.)

**Keywords:** organic electrochemical transistor, OECT, biosensor, glucose, lactate, amplification, layered double hydroxide, LDH

## Abstract

Biosensors based on Organic Electrochemical Transistors (OECTs) are developed for the selective detection of glucose and lactate. The transistor architecture provides signal amplification (gain) with respect to the simple amperometric response. The biosensors are based on a poly(3,4-ethylenedioxythiophene):poly(styrenesulfonate) (PEDOT:PSS) channel and the gate electrode is functionalised with glucose oxidase (GOx) or lactate oxidase (LOx) enzymes, which are immobilised within a Ni/Al Layered Double Hydroxide (LDH) through a one-step electrodeposition procedure. The here-designed OECT architecture allows minimising the required amount of enzyme during electrodeposition. The output signal of the biosensor is the drain current (*I_d_*), which decreases as the analyte concentration increases. In the optimised conditions, the biosensor responds to glucose in the range of 0.1–8.0 mM with a limit of detection (LOD) of 0.02 mM. Two regimes of proportionality are observed. For concentrations lower than 1.0 mM, a linear response is obtained with a mean gain of 360, whereas for concentrations higher than 1.0 mM, *I_d_* is proportional to the logarithm of glucose concentration, with a gain of 220. For lactate detection, the biosensor response is linear in the whole concentration range (0.05–8.0 mM). A LOD of 0.04 mM is reached, with a net gain equal to 400.

## 1. Introduction

In the last 40 years, amperometric biosensors have attracted a great deal of attention due to very appealing features such as fast response, low cost, miniaturisation, and robustness [1,2,3]. A wide range of biologically relevant analytes has been determined through amperometric detection. Among them, glucose [4,5,6,7] and lactate [8,9,10] are crucial target compounds in medicine, sport, nutritional science, and food quality control. The literature offers many examples of amperometric biosensors for glucose and lactate detection, most of them based on the functionalisation with glucose oxidase (GOx) [11] or lactate oxidase (LOx) [12] enzymes. On one hand, the real-time analysis of glucose is generally carried out invasively by subcutaneous electrodes, thus limiting the determination to the most relevant medical cases, such as diabetic patients. On the other hand, lactate determination is usually performed in blood, and real-time data acquisition systems are commercially unavailable, even if some sweat sensors are under development to meet the market demand (http://www.epicorebiosystems.com/). Indeed, other biological fluids such as sweat, saliva, and tears are more readily accessible and thus are attractive targets for non/minimally invasive, portable sensing platforms. Moreover, several quantitative correlations have been recently reported for biomarkers levels in blood and sweat or saliva [13,14,15,16], thus providing the medical relevance of these biofluids. Among the problems that limit the widespread diffusion of portable chemical sensors for non-invasive monitoring of biofluids, the operation of the sensing element is a key issue [17,18], as it must provide a highly sensitive, reproducible and reliable detection, together with significant signal amplification and a simple electronic readout. With a conventional three-electrode cell configuration, an additional component is often required for adequate signal amplification. In this regard, Organic Electrochemical Transistors (OECTs) have the potential to address these requirements. In an OECT sensor, the variation of the input signal, which can be due to redox reactions involving the analyte, typically leads to a modulation of the output signal that is several times higher [19]. Our group has thoroughly investigated the ability of OECTs to amplify faradaic currents, taking as an example the detection of ascorbic acid [20]. On one hand, the transistor amplification enhances the output signal with the consequent sensitivity improvement and decrease of the limit of detection (LOD). On the other hand, the possibility to record a higher output signal, as well as the absence of a reference electrode, leads to the simplification of the readout electronics and allows straightforward miniaturisation of the sensing platform, which is a desirable feature in view of the production of low-cost and portable devices. 

OECTs are three-terminal devices in which the drain and source electrodes are connected by a channel of doped semiconducting polymer (in most cases poly(3,4-ethylenedioxythiophene), PEDOT), and the third electrode (gate) is separated from the channel by means of an electrolyte solution [21,22]. The current flowing through the semiconductor channel (*I_d_*) can be modulated through the application of a potential to the gate electrode (*V_gs_*). In 1985, Wrighton et al. [23] fabricated the first OECT and introduced the idea of electrochemical gating as a tool to control charge transport in a conductive polymer. In the case of a p-type semiconducting channel, the application of a positive *V_gs_* generates a negative polarisation of the channel, thus stimulating the reduction of charge carriers with a consequent decrease of the *I_d_*. If the analytes under investigation take part in the electrochemical processes that control the doping of the semiconducting polymer and modulate the channel conductivity, OECTs can work as chemical sensors. In particular, when a redox reaction involving the analyte occurs at the gate electrode (or at the OECT channel, depending on the applied voltages), it affects the doping level of the transistor channel, resulting in a variation of *I_d_*. The charge transfer process is equivalent to the one of a common amperometric sensor. In principle, any transducer material applied to amperometric sensing could be employed to functionalise the gate electrode of an OECT sensor, with the major advantage that the transistor structure will amplify the recorded signal [20]. As OECTs do not need a reference electrode, which is essential in conventional electrochemical devices, they can be fabricated by means of soft techniques, such as spin coating, roll-to-roll processing, and ink-jet printing, allowing their implementation onto unconventional flexible substrates such as fabrics [24,25,26] or plastics [27,28,29]. Thanks to these features, OECTs stand out as appealing platforms in view of wearable applications and have been used in the last years to realise highly sensitive sensors for a wide range of analytes [30,31,32,33].

While amperometric biosensors based on electrodes modified by GOx or LOx are well established and have been fully studied since 1980, the first paper describing the use of an OECT for glucose analysis was published only 10 years ago and made use of a Pt gate electrode, while GOx was dissolved in the electrolyte solution [34]. Since then, several authors have used OECTs for glucose [19,30,33,35,36] and lactate [36,37,38,39] detection, proposing different strategies to conveniently entrap the enzyme at the gate electrode of the transistor. Since the device performance largely depends on the features of the gate electrode, the best results have been achieved by OECT sensors using Pt nanoparticles. In 2007, our group performed GOx immobilisation on a Pt electrode by means of the co-electrodeposition of the enzyme and a Ni/Al Layered Double Hydroxide (LDH) matrix [40]. The resulting glucose biosensor showed remarkable properties in terms of sensitivity and response time, wide linearity range, long-term stability, and ability of operating in FIA (flow injection analysis) conditions. LDHs are anionic clays with the general formula [M(II)_1−x_M(III)_x_(OH)_2_]^x+^(A^n−^_x/n_)·mH_2_O, where x ranges from 0.22 to 0.33, M is a metal, and A^n−^ is an n- valent anion. Major advantages of using LDHs as enzyme immobilizers include (1) biocompatibility, (2) high mobility of the analyte as well as of the reaction products, and (3) high chemical and hydrolytic stability [41]. Moreover, the one-step electrochemical strategy allowing concurrent enzyme immobilisation and LDH synthesis is particularly appealing for sensors development [40]. First of all, the presence of the LDH on the electrode surface significantly improves the signal-to-noise ratio associated to H_2_O_2_ oxidation, ensuring signal stability in a wide range of concentrations. Moreover, the LDH provides a watery and non-toxic environment, which is desired to preserve the enzyme activity. It is also worth noting that the optimised procedure for enzyme immobilisation is very rapid (30 s) and requires a low amount of enzyme. Indeed, our previous work demonstrated that the achieved enzyme loading is about 50 μg/cm^2^, which is at least one order of magnitude lower than that employed in typical enzymatic biosensors. 

In this work, the procedure proposed in the past for coating Pt electrodes [40,42] was adapted for the modification of the Au gate electrode of a planar OECT, with the aim to make transistor-based biosensors with high sensitivity to glucose and lactate. In particular, the opportunity of using the OECT to amplify faradaic signals is highlighted to design electrochemical sensors and biosensors with improved performance.

## 2. Materials and Methods

### 2.1. Materials

CLEVIOS™ PH 1000 (Heraeus) is a commercial product designed to deposit thin PEDOT:PSS films with high electrical conductivity. Potassium nitrate, dihydrogen phosphate monobasic, glutaraldehyde (25% *w*/*w*, aqueous solution, GA), glucose, glucose oxidase Type VII (179,000 U g^−1^) from Aspergillus Niger (GOx), sodium lactate, lactate oxidase (> 20 U mg^−1^) from Aerococcus Viridans (LOx), aluminum nitrate, nickel nitrate, NAFION^®^ perfluorinated ion exchange resin (5% solution in lower aliphatic alcohols/H_2_O mix, 15–20% H_2_O) and sodium hydroxide were obtained from Sigma-Aldrich. All chemicals were of analytical reagent grade and used as received, without further purification. All aqueous solutions were prepared with doubly distilled (DD) water. 

### 2.2. Instruments

The electrochemical depositions were carried out in a single compartment, three-electrode cell via a potentiostat (CH Instrument 660 C, CH Instruments, Inc., Shanghai, China). All potentials were measured with respect to an aqueous saturated calomel electrode (SCE), a Pt wire was used as the counter electrode, and the gate terminal of the transistor was employed as a working electrode. A bipotentiostat (CH Instrument 900 B Scanning Electrochemical Microscope, CH Instruments, Inc., Shanghai, China) was used to perform the electrical measurements by applying source-drain (*V_ds_*) and source-gate (*V_gs_*) potentials and by measuring the respective currents (*I_d_*, *I_g_*). The source collector was connected to the reference and counter electrode terminals of the bipotentiostat. Gate and drain were connected to the working electrode and the secondary electrode, respectively.

Morphology characterisation and thickness evaluation of the LDH and LDH/GOx modified gate electrodes were carried out in air at room temperature in tapping mode with an atomic force microscopy (AFM) Park System NX10 (Park System, Suwon, Korea).

### 2.3. OECT Sensor Preparation

**Figure 1:**
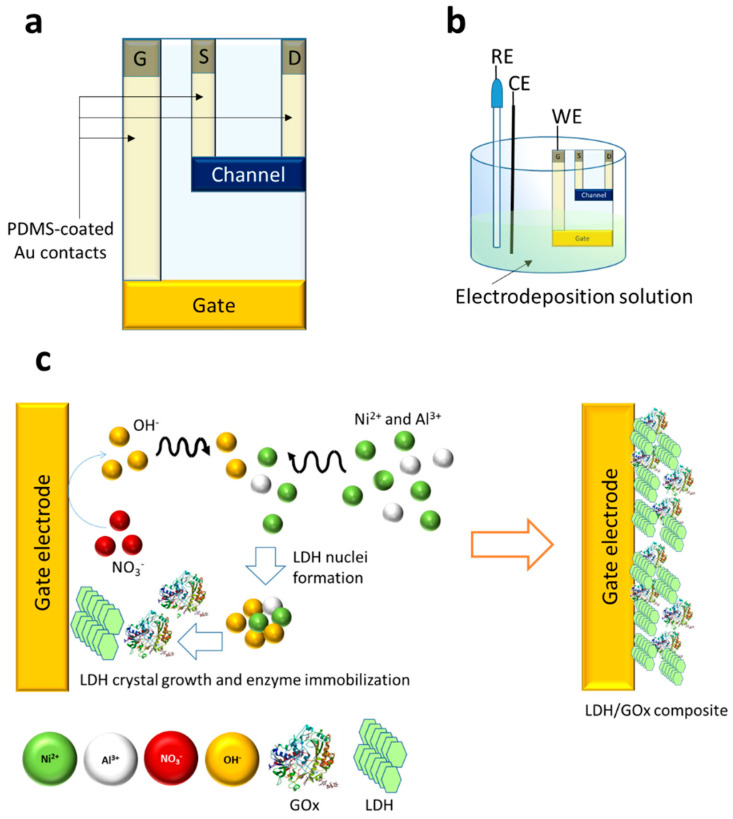
Sensor architecture. Scheme of the device geometry (**a**); experimental setup employed for the gate electrode modification (**b**); processes occurring at the gate electrode, where nitrate reduction induces the increase of OH^−^ concentration, resulting in the precipitation of the Layered Double Hydroxide (LDH)/enzyme composite (**c**).

Cr/Au (50 nm) films were deposited on a cleaned glass slide via thermal evaporation to prepare the gate, drain, and source contacts. The gate electrode is a rectangle with a width of 2.0 cm. The length was varied in order to obtain different ratios of gate-to-channel areas. CLEVIOS^TM^ PH 1000 suspension was mixed with ethylene glycol, 3-glycidyloxypropyltrimethoxysilane, and dodecylbenzene sulfonic acid in the following mass ratio 93.75:5.00:1.00:0.25. The mixture was filtered through a 1.2 μm cellulose acetate filter and spin coated with a rectangular mask (1.0 cm × 0.3 cm) between gold source and drain electrodes (500 rpm, 3 s). The PEDOT:PSS film was annealed at 140 °C for 30 min, and the final thickness of the channel was 800 nm. The OECT geometry is reported in Figure 1. The resulting channel area (A_ch_) was 0.33 ± 0.08 cm^2^, while the gate area (A_g_) was varied to study its effect on the transistor response. The A_g_/A_ch_ area ratios were 2, 4, and 8, for nominal A_g_ of 0.65 ± 0.09, 1.4 ± 0.2, and 2.7 ± 0.2 cm^2^, respectively. Afterwards, the gate electrode of the transistor was modified with the composite Ni/Al LDH/enzyme (GOx or LOx), following the procedure adapted from [40,42] and reported in Figure 1c.

Briefly, 3.0 mL of an aqueous solution containing 0.0225 M Ni(NO_3_)_2_, 0.0075 M Al(NO_3_)_3_, and 0.3 M KNO_3_ were mixed with 0.5 mL of the enzymatic suspension consisting of a 0.1 M PBS solution (pH 7.00) containing 12.5 mg mL^−1^ GOx or 3.9 mg mL^−1^ LOx. The resulting solution was placed in a small volume single compartment cell and a potential of −1.3 V versus SCE was applied to the gate electrode for 35 s. The cathodic potential stimulates the nitrate and water reduction according to reactions (1–6).

H^+^ + e^−^ → H_ads_ (1)

2H^+^ + 2e^−^ → H_2_ (2)

NO_3_^−^ + 2H^+^ + 2e^−^ → NO_2_^−^ + H_2_O (3)

2H_2_O + 2e^−^ → H_2_ + 2OH^−^ (4)

2H_2_O + 2e^−^ → H_2_ + 2OH^−^ (5)

NO_3_^−^ + H_2_O+ 2e^−^ → NO_2_^−^ + 2OH^−^ (6)

The resulting pH increase leads to the precipitation of the layered double hydroxide on the electrode surface.

To prevent the enzyme release, the modified OECTs were placed for 20 min in a saturated atmosphere of GA vapours, in equilibrium with a 25% (*w*/*w*) GA solution. GA has two aldehyde groups that can react with nucleophilic functionalities (such as amine groups) to cross-link proteins matrices by creating intermolecular bonds. 

The same procedure was also employed to coat a 2 mm diameter Au electrode with geometrical area of 0.031 cm^2^. The OECT biosensors were stored at 4 °C in phosphate buffer (pH = 7.00) when not in use. Before utilization, the electrodes were kept at room temperature and soaked into the phosphate buffer solution for 30 min to equilibrate the temperature of the biofilm. For interference studies, 200 µL·cm^−2^ of a Nafion hydroalcoholic solution diluted in water 1:5 were drop-cast on the gate electrodes, and the membranes were dried in an oven at 60 °C. 

## 3. Results

### 3.1. Transistor Configuration and Characterisation

The OECT based on GOx was first developed to optimise the architecture of the device and to find out the best experimental conditions to carry out the detection of the analyte. The electrochemical procedure employed for the biosensor preparation required the design of a dedicated transistor geometry. The OECT configuration of choice (Figure 1a) allows keeping only the gate terminal dipped into the electrolytic solution employed for the enzyme/LDH immobilisation (Figure 1b), thus avoiding the use of a large amount of solution and reducing the device preparation costs. All the contacts, made of gold, were insulated by a polydimethylsiloxane (PDMS) layer that allows tokeep them dry and insulated from the electrolyte.

**Figure 2:**
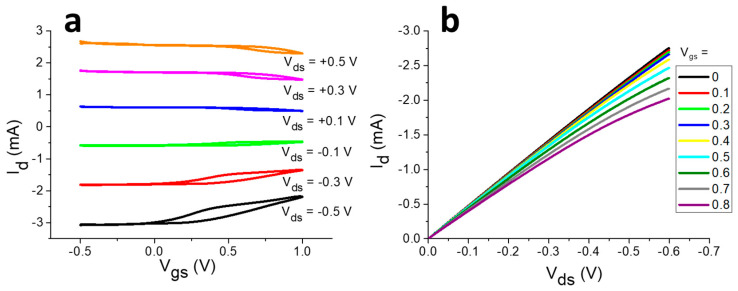
Organic Electrochemical Transistor (OECT) characterisation. Transfer curves recorded for *V_ds_* = −0.5, −0.3, −0.1, +0.1, +0.3, +0.5 V (A_g_/A_ch_ = 4) (**a**); output curves recorded within −0.6 < *V_ds_* < 0 V in 0.1 M phosphate buffer aqueous solution (pH = 7.00) (**b**).

I–V characteristics of the OECT having the geometry just described are reported in Figure 2 and show the typical current modulation upon gating across the electrolyte solution. In the transfer curves (Figure 2a), *V_ds_* is applied between the source and drain collectors to stimulate a current flow through the channel (*I_d_*), and it is chosen to ensure a *I_d_* that is at least one order of magnitude higher than the leakage current between the gate and source. At the same time, *V_gs_*, which is applied with respect to the source terminal, is ramped linearly versus time to induce the electrochemical processes that modulate the current flowing in the channel. When a positive *V_gs_* is applied, the gold gate electrode is positively charged, thus inducing the formation of an electrical double layer in the electrolyte. This originates the flow of a current between the source terminal and the gate terminal (*I_g_*). To close the electrical circuit, the channel/electrolyte interface must be charged. Therefore, the positive ions repelled from the gate electrode enter the conductive polymer and, at the same time, electrons extracted from the gate electrode are injected in the PEDOT:PSS channel, leading to its electrochemical reduction according to the following reaction:

PEDOT:PSS + Na^+^ + e^−^ → PEDOT + Na^+^PSS^−^ (7)

Since PEDOT^+^, which electrostatically interacts with the negatively charged PSS, is the species responsible for charge carriers in the conductive polymer, a decrease of the current that flows in the channel follows Reaction (7). Morphology plays a role on the electrochemical behaviour of commercial PEDOT:PSS, as demonstrated by Volkov et al. [43], who successfully simulated cyclic voltammograms using a two-phase model that simplifies the structure of the conductive polymer to PEDOT-rich and PSS-rich grains. Thanks to Reaction (7), small *V_gs_* variations lead to a high *I_d_* modulation, thus highlighting the transistor amplification from an electronic point of view. When a negative *V_gs_* is applied, the opposite processes take place, and *I_d_* increases. In case of negative *V_gs_*, a small hysteresis appears (Figure 2a) that could be ascribed to the processes involving the formation/destruction of a thin oxide layer on the gold gate electrode. From an electrochemical point of view, the oxide film formation and destruction do not occur at the same potential [44] and this potential offset leads to hysteresis. 

The ability of the device to operate as a transistor can be assessed also by the acquisition of its output characteristics (Figure 2b), where *I_d_* is measured while changing *V_ds_*, and these curves are reported for different *V_gs_* values.

### 3.2. Modification of the Gate Electrode

**Figure 3:**
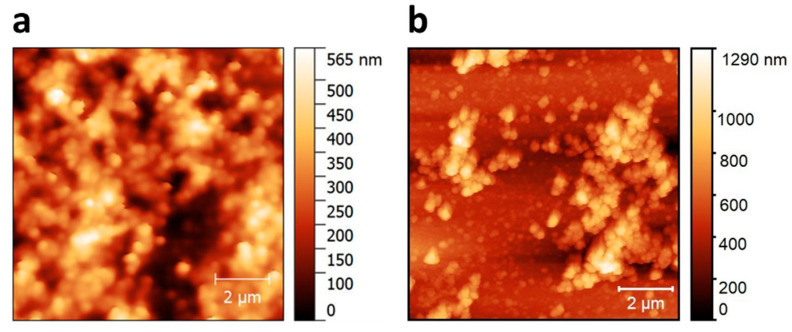
Morphological characterisation. Topography maps obtained by atomic force microscopy (AFM) imaging of gate electrodes modified by electrochemical deposition with Ni/Al LDH (**a**) and Ni/Al LDH and glucose oxidase (GOx) (**b**).

The transduction ability can be obtained in an OECT by the modification of the gate electrode in order to enhance the charge transfer with the target molecules. Figure 1c shows the generation, via the electrodeposition process, of the LDH/GOx composite on the gold gate electrode surface. As described elsewhere [40], the application of a cathodic potential leads to the occurrence of a reaction cascade involving water and nitrate reduction [45], whose overall effect is an increase of the solution pH in close proximity of the electrode surface. This phenomenon leads to LDH/enzyme co-precipitation on the electrode surface, and the final result is the growth of a thin layer of LDH/GOx, which is stabilised by the electrostatic interactions between the positively charged sheets of the LDH and the negative charges of the biomolecule. This procedure, optimised by our group for Pt bulk electrodes, has been here adapted to coat the gate electrode of the transistor, which is made of a thin film of evaporated gold (50 nm thick). It is well known that any electrochemical deposition process is strongly influenced by the nature of the electrode substrate and here, passing from a Pt electrode to an Au thin film, required the optimisation of the electrode-polishing step and of the potential to be applied during the electrode coating. In addition, undesired impurities could be adsorbed on the gold surface during fabrication and must be removed. For the polishing treatment, three different procedures were tested: (1) an electrochemical pretreatment consisting of 100 CV cycles in 0.1 M H_2_SO_4_ in the potential range between −0.25 and +0.6 V versus SCE, (2) immersion in acetone bath for 10 min, and (3) immersion in 0.1 M H_2_SO_4_ bath for 10 min. As regards the applied potential, depositions were carried out at −0.9, −1.0, −1.2 or −1.3 V versus SCE for 35 or 60 s. The best coating quality in terms of homogeneity and full coverage of the gate surface with the LDH/GOx composite was obtained for the electrodes immersed in acetone, as the other procedures lead to a partial degradation of the Au film and thus to a not-uniform gate surface. The best depositions were obtained when the most cathodic potential (i.e., −1.3 V versus SCE) was applied for 35 s. Figure 3 shows AFM images of the gate electrode coated with the Ni/Al LDH film (a) and with the composite LDH/GOx (b). The surface roughness increases for the sample containing the enzyme, being (34 ± 4) nm in the former case and (80 ± 10) nm in the presence of GOx. This result is in agreement with our previous findings [40] and confirms the entrapment of GOx within the LDH film. In both cases, the morphological characterisation reveals the round particles typical of LDH structures and suggests that the enzyme distribution is not homogeneous over the whole electrode surface, as it reflects the distribution of the underlying electrosynthesised inorganic film [46].

Before examining the behaviour of the OECT biosensor, we recall the response expected for a common gold electrode coated by the LDH/GOx film. GOx entrapped in the LDH catalyzes the reaction between glucose and oxygen to give gluconolactone and hydrogen peroxide, according to the following reaction:

Glucose + O_2_ → Gluconolactone + H_2_O_2_. (8)

If a positive potential (+0.45 V vs. SCE) is applied through a potentiostat to the gold electrode in a conventional three-electrode electrochemical cell, H_2_O_2_ undergoes electrochemical oxidation and the recorded current linearly depends on glucose concentration (see Figure S1, wherein the chronoamperometric response of a gold disk electrode coated by GOx/LDH is shown). 

### 3.3. OECT Glucose Sensor

**Figure 4:**
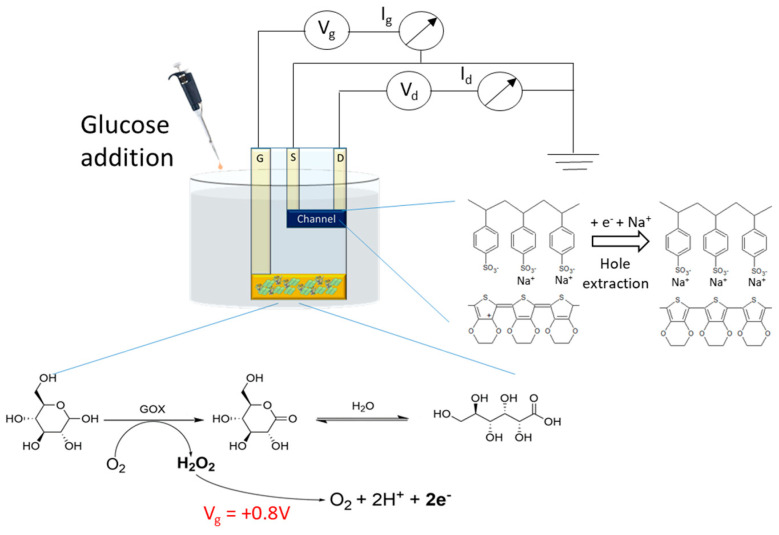
Transduction mechanism. Experimental set up employed for glucose detection at the OECT and chemical and electrochemical reactions occurring at the gate electrode and at the channel during glucose sensing.

The gate modification was performed in order to provide the OECT with glucose-sensing capability. The device response to glucose was assessed in a 0.1 M PBS (pH = 7.00), applying fixed gate and drain potentials (*V_gs_* = +0.8 V; *V_ds_* = +0.1 V). In these conditions, the positive *V_gs_* promotes the electro-oxidation of the in situ produced H_2_O_2_ (Figure 4).

**Figure 5:**
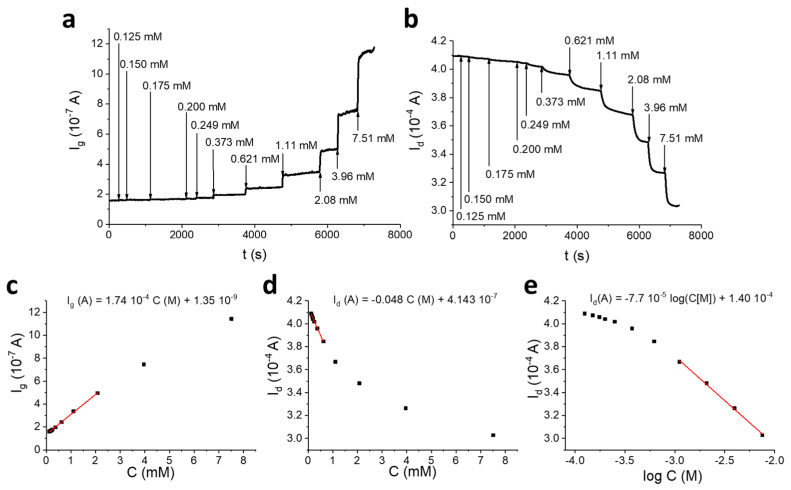
OECT biosensor for glucose detection. *I_g_* (**a**) and *I_d_* (**b**) vs. time curves obtained for the OECT with A_g_/A_ch_ = 4, following the addition of different glucose amounts (*V_gs_* = +0.8 V; *V_ds_* = +0.1 V) in 0.1 M phosphate buffer solution (PBS, pH = 7.00). The additions are indicated with an arrow. Plots of *I_g_* (**c**) and *I_d_* (**d**) as a function of glucose concentration and plot of *I_d_* as a function of the logarithm of glucose concentration (**e**).

Figure 5 shows typical *I_g_* and *I_d_* variations following glucose additions, which were recorded with a device having an A_g_/A_ch_ (gate area/channel area) ratio equal to 4, which has been chosen as it ensures optimised performances to the transistor, as discussed later (for other examined A_g_/A_ch_ values, see Supplementary Materials). In these conditions, a positive *I_g_* flows between the gate and the channel across the electrolyte solution, whose intensity increases as glucose concentration increases (Figure 5a). *I_g_* value is proportional to glucose concentration with a sensitivity of 0.12 mA M^−1^cm^−2^ in the range of 0.1–4.0 mM (see the calibration plot reported in Figure 5c). This response is analogous to the one that would be measured with a conventional amperometric biosensor. 

Since an OECT is not endowed with a counter electrode, H_2_O_2_ electro-oxidation at the gate electrode must be coupled to a reduction process that takes place at the channel. Consequently, the electrons produced by H_2_O_2_ reduce PEDOT:PSS with an extraction of the charge carriers, which leads to a *I_d_* decrease (Figure 5b). Since the electrons taken out at the gate are injected in the channel, the rate of H_2_O_2_ oxidation expressed as a current (*I_g_*) must be the same of PEDOT reduction (i.e., hole extraction). A stationary state *I_d_* and, thus, a stable signal is reached when the rate of holes extraction at the gold/PEDOT interface equals the rate of holes regeneration at the PEDOT/electrolyte interface due to the reaction between PEDOT:PSS and some species in solution. The charge transfer between PEDOT and the electrolyte must occur in order to close the circuit between the source and the gate electrodes. It can be considered as the process that takes place at the counter electrode in a common three-electrode cell to allow the occurrence of the redox process at the working electrode. 

Therefore, *I_g_* and *I_d_* variations (Δ*I_g_* and Δ*I_d_*) can be associated to different processes occurring in the channel. Δ*I_g_* reflects the amount per time unit of holes extracted from the channel following the occurrence of the redox sequence starting from glucose oxidation at the gate electrode. Δ*I_d_* is the current variation measured at the drain that is generated by the extraction of holes from the channel. The hole extraction process generates a higher signal variation if *I_d_* is considered, as demonstrated by ∣Δ*I_d_*∣ > ∣Δ*I_g_*∣. This suggests that each hole that is reduced in the PEDOT:PSS channel due to glucose sensing would travel across the channel many times before its regeneration. Considering that *I_d_* is the OECT amplified signal and *I_g_* is the current due to glucose oxidation, the gain obtained with respect to the simple faradaic signal can be quantified by the ratio between *I_d_* and *I_g_* variations (Gain = ∆*I_d_*/∆*I_g_*), as already described in our previous work [20].

Figure 5d,e show the *I_d_* versus glucose concentration and logarithm of glucose concentration plots, respectively. Two different regimes of proportionality can be observed. For concentrations lower than 1.0 mM, *I_d_* is linearly proportional to glucose concentration (sensitivity: 0.048 A M^−1^), whereas for concentrations ranging between 1.0 and 8.0 mM, *I_d_* is proportional to the logarithm of glucose concentration (sensitivity: 7.7 × 10^−5^ A decade^−1^). The mean response time was 50 s in the optimised conditions. The double regime has already been observed in the literature [47], but the logarithmic response only is explained by the quantitative model developed for OECTs chemical sensing [34]. We are carrying out some additional studies to explain this behaviour because the linear regime exhibits higher sensitivities than the logarithmic one that is widely studied in literature. 

The gain was calculated using three devices with the same A_g_/A_ch_ for both the linear and the logarithmic region and resulted in values of 360 and 220, respectively.

Different devices were tested changing the ratio between the gate and the channel areas, in order to define the geometry ensuring the best signal amplification. On one hand, a high gate area is desirable to generate high *I_g_* following glucose oxidation, but, on the other hand, previous studies have shown that the transistor architecture with low A_g_/A_ch_ guarantees the best sensing performance in terms of amplification [19,48]. For this reason, preliminary experiments were carried out with A_g_ < A_ch_, but the OECT detected glucose very slowly and with a low sensitivity. Therefore, devices having A_g_/A_ch_ equal to 2, 4, and 8 were tested and compared evaluating the detection limit (LOD) and Gain. The increase of the gate area leads to an increase of both the immobilised GOx and the gate capacitance (*C_g_*). As reported by Cicoira et al. [48], high *C_g_* values lead to a lower potential drop at the gate/electrolyte interface and, consequently, the gate action is lower. In fact, the normalised sensitivity values calculated from *I_g_* calibration curves clearly show this effect, as their values decrease upon the increase of A_g_, thus suggesting that the best performance should result from a compromise among the different parameters that control the sensor operation. It is worth noting that when the gate action is maximised (A_g_/A_ch_ = 2), the transistor exhibits an *I_g_* sensitivity that equals the amperometric sensor. 

Table 1 shows the mean results obtained from the screening experiments (N = 3). For a device having an A_g_/A_ch_ ratio equal to 8, the linear region cannot be defined, being *I_d_* linearly related to the logarithm of glucose concentration in the whole concentration range. 

The best LOD value is observed for an A_g_/A_ch_ ratio equal to 4, highlighting that the optimisation of the OECT sensor performance means finding a compromise between transduction ability and transistor amplification. If compared to the amperometric biosensor, the LOD achieved by the OECT sensor is more than 10 times lower (0.02 versus 0.3 mM) due to the intrinsic signal amplification provided by the transistor architecture. Overall, papers concerning OECT sensors rarely show the absolute values of drain and gate currents and do not point out the amplification of the faradic signal. To this regard, the gain observed for our devices is comparable with the only one that can be calculated from literature data [19], the results of which are equal to 150. Among other OECT biosensors for glucose detection, the performance of the here reported device is lower than those obtained by OECTs functionalised with Pt NPs or TiO_2_ nanotubes [30,33,35]. Nevertheless, the operative range of our sensor is wider than those of ferrocene-modified OECT sensors [36,49]. While the linear sensor response is suited for salivary glucose testing and covers the cut-off range associated to potential type 2 diabetes mellitus [16], the logarithmic region is sufficient for screening normal glucose concentrations in blood [7].

Redox active compounds can affect the OECT operation due to charge transfer processes occurring at the gate or at the channel. Here, the effect of ascorbic acid (AA), uric acid (UA), and lactate presence was studied on the response of either the as-prepared device or after gate modification with a Nafion membrane, in order to hinder the diffusion of the negatively charged interferents to the gate electrode. Random additions of AA, UA, and lactate within typical concentration ranges found in biofluids such as saliva [50,51,52] were made to the electrolyte solution during *I_d_*/time recordings. In order to evaluate their effect on the sensor response during glucose detection, we used the formula (I_glu_ − I_int_)/I_glu_ × 100, where I_glu_ and I_int_ are the steady-state drain currents recorded after a control glucose addition and subsequent interfering species additions, respectively. If the device is used as prepared, +38 and +45% variations of *I_d_* are observed due to UA and AA, respectively, while lactate addition does not produce any current change. Differently, the use of a Nafion membrane drastically improves the sensor selectivity (Figure S4), as the interference of AA is reduced to +(7.0 ± 0.8)% (N = 3), and no current variation is recorded following UA addition. 

Finally, the stability and reproducibility of the OECT sensor were evaluated. The reproducibility was investigated considering the normalised current response (1 − I/I_max_, where I_max_ is the drain current at the beginning of analysis, and I is the drain current to be normalised). Figure S5 shows the response of three different sensors having A_g_/A_ch_ equal to 4, highlighting a satisfactory reproducibility. As regards stability, the sensor response was evaluated during a 10-day utilization period, keeping the OECT at 4 °C soaked in 0.1 M PBS when not use. Each day, two calibration graphs were recorded within the linearity range, one at the beginning and one at the end of the day, and the average slope was calculated. The OECT retained its initial activity for 5 days and 70% of the initial activity was still retained after 10 days. The intraday repeatability of the sensor response was evaluated by repeating a complete calibration curves three times in one day, and a variation of the slope of the curve lower than 5% was obtained.

### 3.4. OECT Lactate Sensor

**Figure 6:**
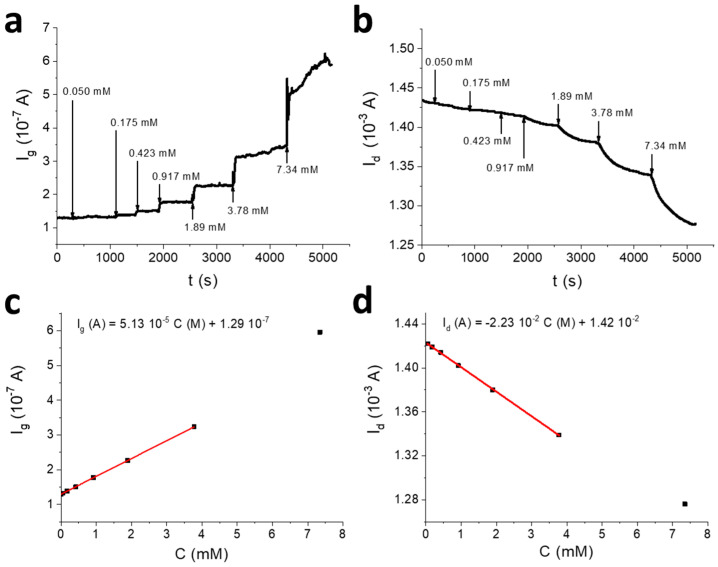
OECT biosensor for lactate detection. *I_g_* (**a**) and *I_d_* (**b**) vs. time curves obtained for the OECT sensor with A_g_/A_ch_ = 4, after the addition of increasing lactate amounts (*V_gs_* = +0.8 V; *V_ds_* = +0.1 V) in 0.1 M PBS (pH = 7.00). The additions are indicated with an arrow. Plot of *I_g_* (**c**) and *I_d_* (**d**) as a function of lactate concentration.

One of the main advantages of the procedure here employed to immobilise GOx on the gate electrode is that it can be applied to any enzyme. To demonstrate that, the electrodeposition procedure optimised for GOx was further exploited for immobilising lactate oxidase on the gate electrode of an OECT with previously optimised geometry (A_g_/A_ch_ = 4). The enzyme immobilisation was achieved by applying a potential of −1.3 V versus SCE to the gate electrode for 35 s using the experimental setup depicted in Figure 1,b. The OECT response to lactate was then assessed in 0.1 M PBS (pH = 7.00), in the same experimental conditions used for glucose (*V_gs_* = +0.8 V and *V_ds_* = +0.1 V). *I_g_* and *I_d_* vs. time curves following lactate additions, as well as the corresponding calibration plots, are shown in Figure 6. 

It is worth noting that *I_d_* is linearly proportional to lactate concentration in the whole concentration range (sensitivity: 2.23 × 10^−2^ A M^−1^), no logarithmic dependence being detected. An average gain of 400 was calculated in this case, and the LOD that resulted was equal to 0.04 mM. Despite the simpler architecture, the here-described sensor is comparable to most of the OECT-based sensors for lactate detection reported in the literature in terms of sensing performance [36,37,38,39]. The wide linear range of the OECT sensor is compatible with normal lactate range in blood serum [53,54] and salivary lactate testing at rest and during physical activity [15,52].

## 4. Conclusions

In this work, two OECT-based biosensors were developed for the analysis of glucose and lactate. For enzymes’ immobilisation, a simple, rapid, and reproducible procedure was employed, based on the one-step electrochemical co-deposition of GOx/LOx and Ni/Al LDH on the gate electrode of the OECT. The LDH acts both as an immobilisation matrix and as an electrode modifier, allowing for a stable signal to be recorded following the oxidation of the enzymatically produced H_2_O_2_. The procedure of choice for the electrochemical functionalisation of the Au gate electrode is versatile and can be used to immobilise enzymes of different nature. The design of a dedicated OECT architecture was required so that the gate electrode can operate as a working electrode in a small volume, three electrode cell, thus minimising the required amount of enzyme that usually most impacts on the overall device cost. The OECT geometry was optimised based on glucose detection performance, and our results show that A_g_/A_ch_ = 4 guarantees the best performance in terms of limit of detection and amplification. 

In OECT biosensors based on oxidases, if the gate electrode is polarised at potentials higher than the channel, the enzymatically produced H_2_O_2_ undergoes oxidation at the gate terminal, leading to an increase of *I_g_* and to a decrease of *I_d_*. The sensor gain is a useful figure of merit to evaluate the performance of transistor-based sensors with respect to the consolidated amperometric counterparts. It is estimated by the ratio between *I_g_* and *I_d_* variations, the first term being analogue to the signal that would be measured using an amperometric biosensor. 

For glucose detection, two regimes of proportionality can be observed for *I_d_*, and the higher sensitivity observed at low glucose concentrations allows reaching a very low limit of detection (0.02 mM), which is at least 10 times lower than a LOD displayed by an amperometric biosensor endowed with a working electrode prepared in the same way as the gate terminal. Since the linear response exhibits a higher gain, we are studying this sensing regime to improve the OECT performance. 

For lactate detection, *I_d_* is linearly proportional to the concentration in the whole investigated concentration range. The LOD is 0.04 mM, and despite the simpler architecture, the device is comparable to most of the OECT-based sensors for lactate reported in the literature in terms of sensing performance. The here-proposed biosensors could be employed for the non-invasive screening of glucose and lactate in biofluids such as saliva, where typical concentrations lie in the ranges of 0.1–0.5 mM and 0.1–2.5 mM, respectively. 

## Figures and Tables

**Table 1:** Limit of Detection (lod), Sensitivities, and Average Gain in the Linear (“lin”) and Logarithmic (“log”) Regions Obtained for Oects Having Different a_g_/a_ch_ Values.

A_g_/A_ch_	LOD (mM)	*I_g_* Sensitivity (mA M^−1^ cm^−2^)	*I_d_* Sensitivity^lin^ (mA M^−1^ cm^−2^)	Gain^lin^	*I_d_* Sensitivity^log^ (mA decade^−1^ cm^−2^)	Gain^log^
2	0.15	0.18 ± 0.04	64 ± 4	360	0.10 ± 0.05	160
4	0.02	0.12 ± 0.02	43 ± 5	360	0.043 ± 0.008	220
8	0.03	0.036 ± 0.008	N/E	N/E	0.024 ± 0.005	120
Amperometric Sensor	0.3	0.18 ± 0.03*	N/A	N/A	N/A	N/A

*Sensitivity of the amperometric sensor of Figure S1. N/E not estimable; N/A not applicable.

## Supplementary Materials

The following are available online at https://www.mdpi.com/1424-8220/20/12/3453/s1. Figure S1: Response of a glucose amperometric biosensor, Figure S2: OECT biosensor for glucose detection with A_g_/A_ch_ = 2, Figure S3: OECT biosensor for glucose detection with A_g_/A_ch_ = 8, Figure S4: Response of the Nafion modified OECT biosensor, Figure S5: Reproducibility of the OECT biosensor.
