# Supplementary material for: Layered Double Hydroxide-Modified Organic Electrochemical Transistor for Glucose and Lactate Biosensing
Source: Sensors (Basel). 2020 Jun 18;20(12):3453. doi: 10.3390/s20123453 (PMC7348845; doi:10.3390/s20123453)
Supplement: Supplementary file 1 [file sensors-20-03453-s001.pdf]

Supplementary Materials

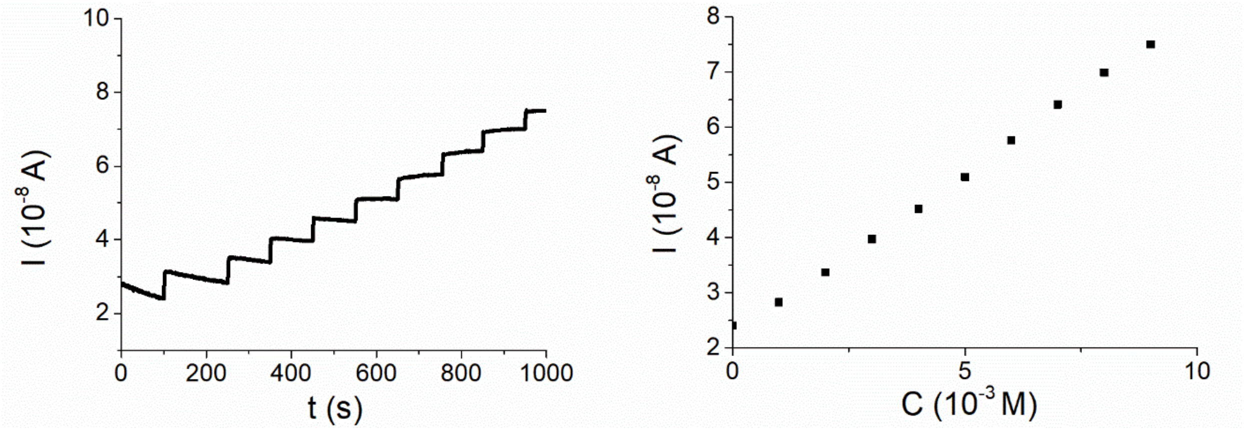

Figure S1. Response of a glucose amperometric biosensor. Chronoamperometric curves (**left**) and calibration plot (**right**) recorded at a gold disk electrode (diameter = 0.200 cm) coated with a LDH/GOx film in 0.1 M phosphate buffer solution (pH = 7.00) during 1.00 mM glucose additions.

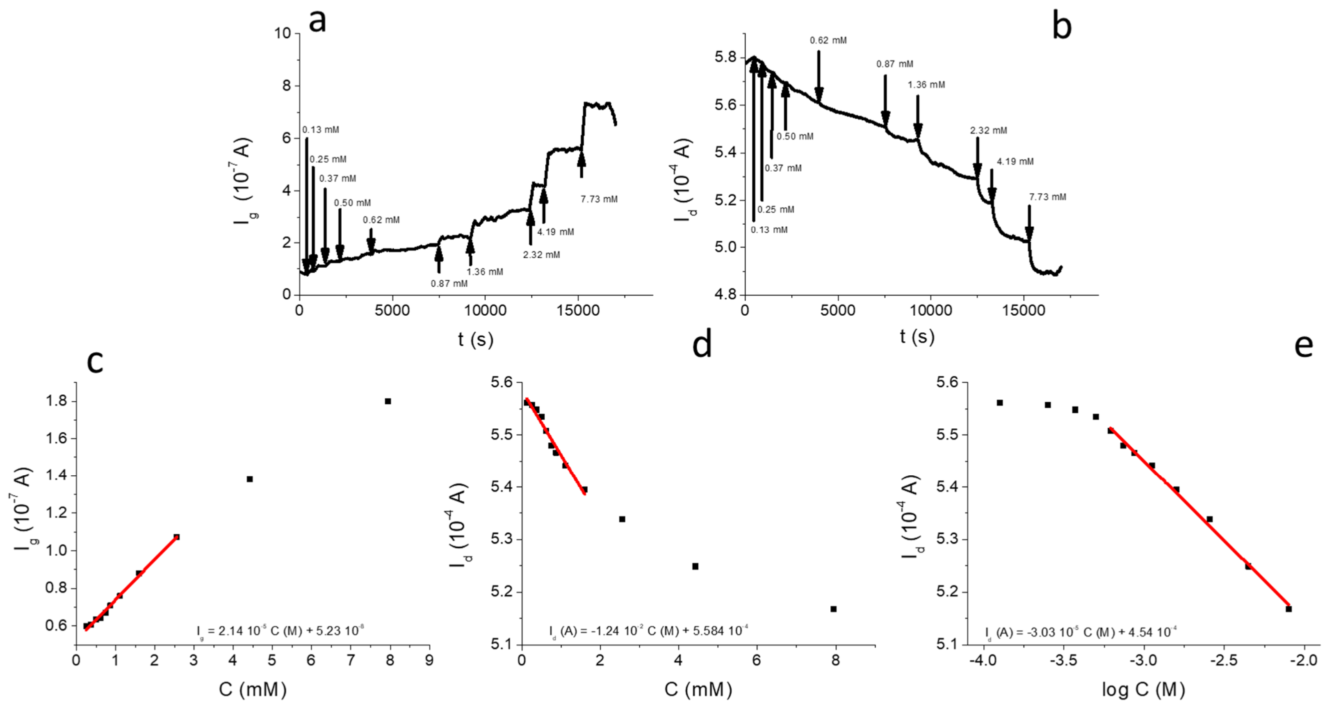

Figure S2. OECT biosensor for glucose detection with A_g/A_{ch}= 2. I_g (**a**) and I_d (**b**) vs time curves obtained for the OECT with A_g/A_{ch}= 2, following the addition of different glucose amounts (V_{gs} = +0.8 V; V_{ds} = +0.1 V) in 0.1 M aqueous PBS (pH = 7.00). The additions are indicated with an arrow. Plots of I_g (**c**) and I_d (**d**) as a function of glucose concentration and plot of I_d as a function of the logarithm of glucose concentration (**e**).

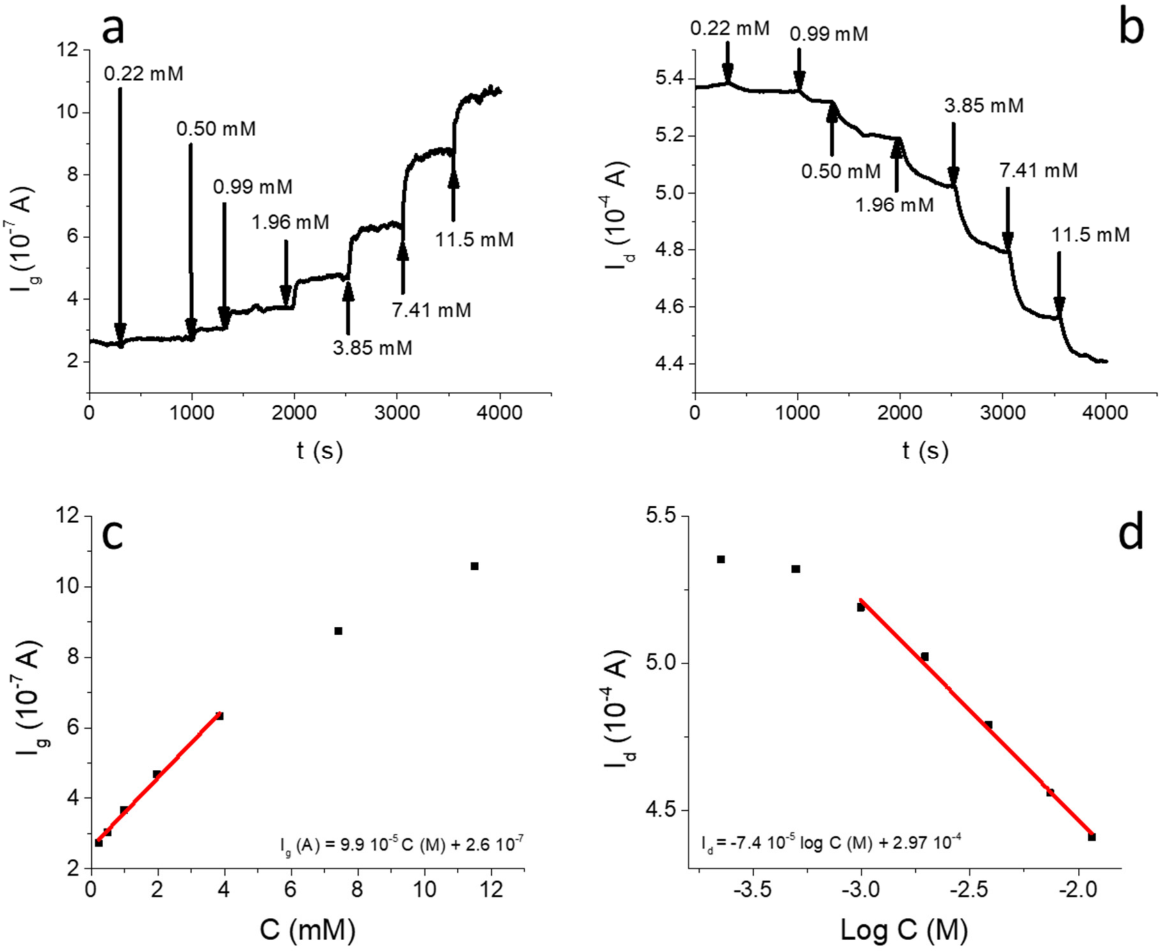

Figure S3. OECT biosensor for glucose detection with A_g/A_{ch}= 8. I_g (**a**) and I_d (**b**) vs time curves obtained for the OECT with A_g/A_{ch}= 8, following the addition of different glucose amounts (V_{gs} = +0.8 V; V_{ds} = +0.1 V) in 0.1 M aqueous PBS (pH = 7.00). The additions are indicated with an arrow. Plots of I_g as a function of glucose concentration (**c**) and plot of I_d as a function of the logarithm of glucose concentration (**d**).

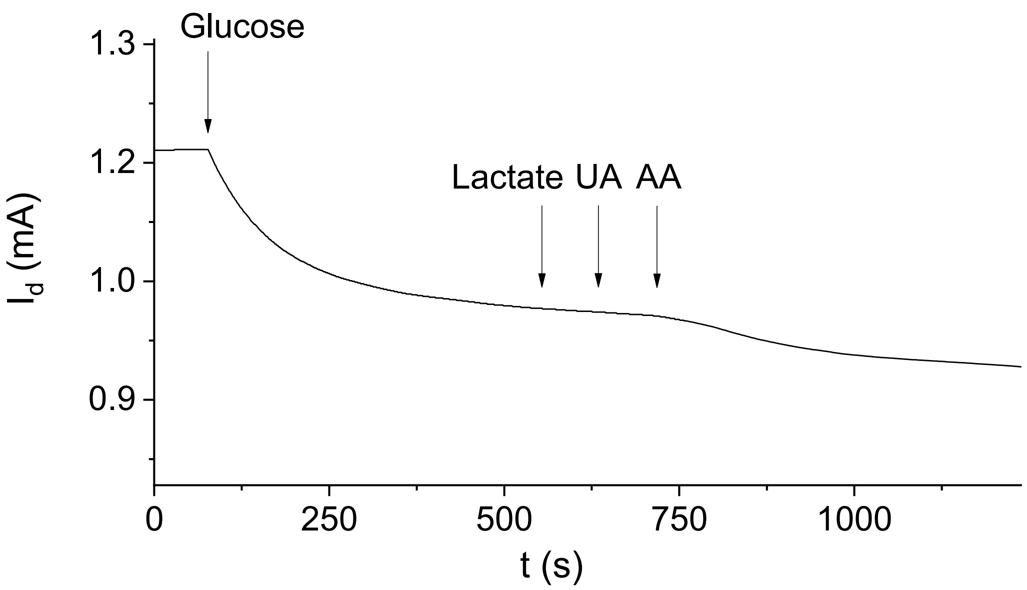

Figure S4. Response of the Nafion modified OECT biosensor. I_d vs time curve (V_g = +0.8 V; V_d = +0.1 V) obtained for the OECT biosensor with A_g/A_{ch}= 4, following a control addition of glucose (4.76 mM) and additions of 5.00 mM lactate, 0.05 mM uric acid (UA) and 0.05 mM ascorbic acid (AA) in 0.1 M PBS (pH = 7.00).

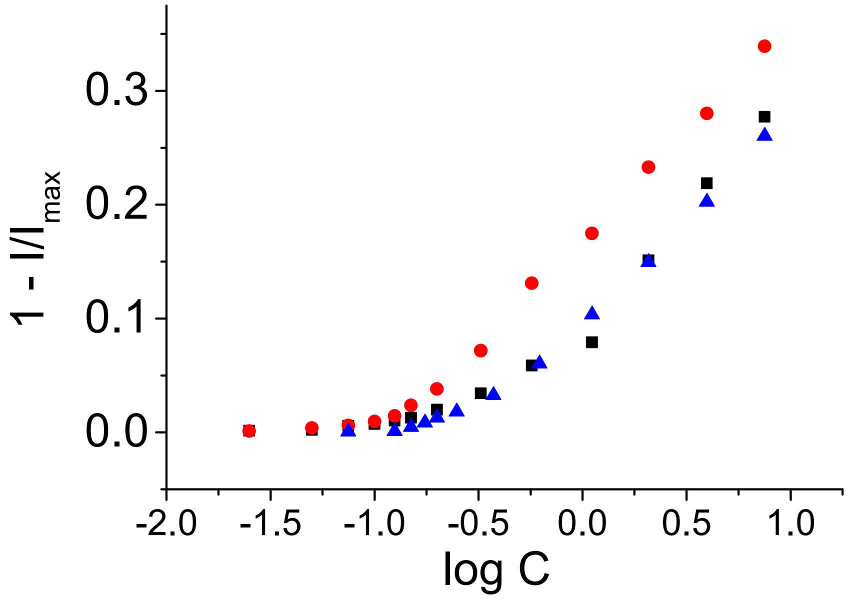

Figure S5. Reproducibility of the OECT biosensor. Normalized response to glucose for three different OECT sensors having A_g/A_{ch}= 4 in 0.1 M PBS (pH = 7.00).
